# Effect of dietary supplementation with zinc-methionine on ruminal enzyme activities, fermentation characteristics, methane production, and nutrient digestibility: An *in vitro* study

**DOI:** 10.5455/javar.2023.j725

**Published:** 2023-12-31

**Authors:** Moh. Sofi’ul Anam, Andriyani Astuti, Budi Prasetyo Widyobroto, Ali Agus

**Affiliations:** Department of Animal Nutrition and Feed Science, Faculty of Animal Science, Universitas Gadjah Mada, Yogyakarta, Indonesia

**Keywords:** Enzyme activities, *in vitro* technique, nutrient digestibility, zinc-methionine, rumen fermentation characteristics

## Abstract

**Objective:**

The principal objective of this research was to examine the influence of zinc-methionine (Zn-Met) addition on feed on the *in vitro* ruminal enzyme activities, fermentation characteristics, methane production, and digestibilities of feed nutrients.

**Materials and Methods:**

The dosage of Zn-Met as a source of organic Zn was added to feed based on dry matter (DM) as follows: 0-without Zn addition (CON), 30 Zn mg/kg-low (LZM), 60 Zn mg/kg-medium (MZM), and 90 Zn mg/kg-high (HZM).

**Results:**

The results indicated significant impacts of Zn-Met addition on various parameters. Compared to the CON group, all Zn-Met treatments resulted in increased total volatile fatty acids (VFA) (linear; *p* < 0.001), carboxymethyl-cellulase activity (linear; *p* < 0.001), and gas production at 48-h of incubation (linear; *p* < 0.001, quadratic; *p* < 0.001). Additionally, the MZM and HZM groups exhibited higher levels of amylase activity (linear; *p* < 0.001), protease activity (linear; *p* = 0.006), microbial protein (linear; *p* = 0.009), DM digestibility (linear; *p* < 0.001), organic matter (OM) digestibility (linear; *p* < 0.001), crude protein (CP) digestibility (linear; *p* = 0.004), and crude fiber (CF) digestibility (linear; *p* = 0.003) than CON treatment. However, the treatments did not have any noteworthy effects on pH, the individual VFA (acetate, propionate, and butyrate) proportions, NH_3_-N concentration, and methane production (*p* > 0.05).

**Conclusion:**

It could be summarized that supplementing 60 and 90 Zn mg/kg DM as Zn-Met could improve the *in vitro* ruminal enzyme activities, fermentation characteristics, and nutrient digestibility without affecting methane production.

## Introduction

Trace minerals, including zinc (Zn), play essential roles in animal feed due to their physiological functions, impact on immune and reproductive systems, and overall animal performance [1–1]. Zn, in particular, is involved in crucial physiological processes such as cell division, proliferation, deoxyribonucleic acid (DNA) synthesis, gene expression, and enzyme cycles. Furthermore, Zn is imperative for ruminant feed and metabolic functions of rumen microorganisms [4].

Zn deficiency is commonly observed in rumen microbial growth, which can limit feed degradation. Adequate levels of Zn are crucial for maximizing the ruminal feed substrate digestion and promoting microbial growth, as Zn serves as an activator for numerous enzymes [5]. Hence, it is strongly advised to provide trace mineral supplements in regions where forage alone cannot meet the mineral needs. This is evident because only 2.5% of 352 forage samples contained sufficient Zn [6]. Similarly, livestock feed in tropical regions like Indonesia mostly contains low levels of Zn [7]. The suggested dietary Zn dosage aligning with worldwide animal requirements for ruminant animals is around 50 mg/kg dry matter (DM) [8]. Supplementation of Zn in practical diets improved rumen fermentation characteristics, digestibility of nutrient content, and livestock performance [9]. Li et al. [10] also observed diminished methane (CH_4_) emissions and reduced populations of methanogens in lactating cows after introducing mineral salt supplements to their diet. Dietary mineral salt led to a decline in CH_4_ emissions by altering methanogen characteristics, modifying the acetate (C_2_) to propionate (C_3_) ratio, and enhancing the utilization of hydrogen ions in C_2_ synthesis.

Traditionally, inorganic sources provide dietary supplementation of Zn, such as Zn oxide and Zn sulfate. However, organic mineral sources have garnered attention due to their superior bioavailability and potential benefits for animals. Trace elements in organic form, bound to organic ligands, are expected to exhibit more excellent resistance and potentially be more bioavailable in the digestive tract of ruminants than inorganic sources [11]. Enhancing the retention and absorption efficiency of organic Zn supplementation could mitigate the adverse environmental effects of animal Zn excretion [12]. The exploration of using Zn-amino acid chelates as animal feed supplements, such as Zn-methionine (Zn-Met), has increased in recent years. Nevertheless, the optimal dosage of Zn-Met supplementation remains less defined. Chen et al. [13] suggested that supplementing prepartum cows with 40 Zn-Met mg/kg of DM may improve the passive transfer of immunity.

Nevertheless, findings on the effects of dietary Zn-Met on enzymatic activity and methane emissions in the rumen still need to be made available. Additionally, research on organic Zn in tropical countries, especially Indonesia, still needs to be available. Thus, this current research aims to explore the impacts of Zn-Met dosage addition on feed, as an organic Zn source, on ruminal enzyme activity, fermentation characteristics, methane production, and digestibility of nutrient content through an *in vitro* approach.

## Materials and Methods

### Sample preparation and research design

The research methodologies employed in the study were ethically accepted by the Ethical Commission for Animal Research (Universitas Gadjah Mada, Indonesia) under the number 025/EC-FKH/Eks./2023. The diet’s substrate was made by combining 60% DM elephant grass, 40% DM wheat bran, and a 0.5% mineral blend top-up. This composition is representative of standard farming practices in Indonesia. The fresh elephant grass was chopped and dried at 55°C for 3–4 days. Subsequently, it was milled utilizing a Willey Mill. Proximate analysis was used to determine the nutrient content of the feed samples [14], while the Zn content was analyzed using atomic absorption spectroscopy (AAS). The nutrient compositions of the basal diet were as follows: 88.06% DM, 87.89% OM, 12.69% CP, 1.91% EE, 23.86% CF, 61.11% total digestible nutrient (TDN), and Zn content in the diet was 22.03 mg/kg DM.

This trial had a fully randomized design. The treatments included a basal substrate without addition of Zn-Met (CON), Zn-Met addition at 30 mg Zn per kg DM, defined to as low Zn-Met (LZM), Zn-Met addition at 60 mg Zn per kg DM, defined to as medium Zn-Met (MZM), and Zn-Met addition at 90 mg Zn per kg DM, defined to as high Zn-Met (HZM). The Zn-Met utilized in this investigation was purchased from PT. Fenanza Putra Perkasa, an Indonesian commercial enterprise. It had a Zn concentration of 15% and a heavy metal level of less than 0.4 ppm.

### Source of rumen liqour

The adaptation treatment of the feed was carried out on two Bali cattle weighing approximately 324 kg of body weight (BW), which served as donors for rumen fluid. During the 7-day adaptation period, the animals were given a concentrate feed containing 13% CP and 75% TDN. Additionally, the cattle were provided with grass *ad libitum*, kept individually, and given unlimited access to water. The purpose of the adaptation period was to standardize the rumen fluid composition by allowing the rumen microbes to adapt to the feed.

A 100 ml glass syringe of the Fortuna model, manufactured by Poulten and Graft GmbH Germany, was filled with 300 mg DM of feed substrate and Zn-Met according to the treatment. Upon completion, the syringes were incubated overnight at 39°C so their contents might appropriately react under controlled thermal conditions until morning [15]. The next day, fresh fluid from the rumen was obtained from the two animals using a cannula immediately before the cattle‘s feeding. Equal amounts of the rumen fluid and its contents were collected from each cattle and transferred into warm thermos bottles. The fluid was then filtered using a double layer of thin commercial filter cloth to remove any coarse particles or solid materials in the liquid. Rumen liquid was added to the media buffer in a 1:2 ratio (v/v). Each sample syringe was filled with a 30 ml mixture of rumen buffer solution, and the syringes were flushed with CO_2_ to guarantee anaerobic conditions. After preparation, each syringe was left to incubate at 39°C for 48 h.

### Gas production and ruminal fluid sampling

Gas production measurements were taken at various time points, including hours 0, 2, 4, 6, 8, 12, 24, 36, and 48. After the 48-h observation period, gas samples were transferred into plain 10 ml vacutainers from the incubated syringes for subsequent CH_4_ content analysis. The determination of CH_4_ was done using a Shimadzu GC-14B gas chromatograph. The fermented fluid from the syringes was also carefully transferred into 20 ml tubes. The rumen fluid underwent centrifugation at 3,000 g for a duration of 15 min.

### Ruminal fluid analysis

The Hanna Model H1–2210 pH meter was employed to measure the rumen fluid’s pH. The activity of carboxyl methyl cellulase (CMC-ase) and amylase enzymes was evaluated depending on the substrate degradation methods. The protease enzyme activity was assessed to determine the protease enzyme’s capability to break down casein into peptides and amino acids. The ruminal VFA was quantified using gas chromatography. The ammonia-nitrogen (NH_3_-N) level was assessed using spectrophotometry. The microbial protein quantification was performed using the Lowry method [16].

### In vitro nutrient digestibility

The digestibilities of specific components, including DM, OM, CP, and CF, were evaluated. The feed substrate was prepared in tubes and mixed with McDougall’s buffer and rumen liquid (4:1 ratio). The sample tubes were rinsed with CO_2_ to ensure anaerobic conditions and then taken into an incubator that operated at 39°C over 48 h. After incubation, a glass wool crucible separated the fermented liquid and the feed substrate. The residue from the separation process was used to measure the ruminal digestibility of DM, OM, CP, and CF.

### Data analysis

The MIXED procedure was used to analyze the data using SAS On Demand for Academics^®^ Software, an open-source statistical software (www.sas.com). The feed treatment served as the fixed effect in the statistical model. Orthogonal contrast was applied to investigate Zn-Met supplementation’s linear and quadratic impacts. Duncan’s multiple range test was implemented to analyze group mean differences at the level of significance, *p* < 0.05. PCA was conducted using JMP 12.0, a statistical software developed by SAS Institute Inc. in Cary, North Carolina, USA.

## Results

### Ruminal enzyme activities

**Figure 1.**
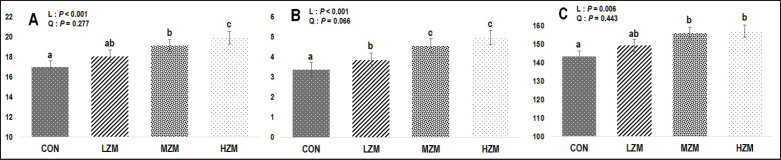
Ruminal enzyme activities of Zn-Met supplementation. (A) Amylase enzyme activity (U/g). (B) Carboxymethyl-cellulase enzyme activity (U/g). (C) Protease enzyme activity (U/g). CON: a basal diet without Zn-Met supplementation, LZM: low Zn-Met (30 mg Zn/kg DM, MZM: medium Zn-Met (60 mg Zn/kg DM), HZM: high Zn-Met (90 mg Zn/kg DM), L: linear contrast, and Q: quadratic contrast. Superscript letters on the figure indicate statistically significant differences at a significance level of *p* < 0.05.

The results of ruminal enzyme activities are depicted in Figure 1. The amylase levels exhibited a linear enhancement (*p* < 0.001) with the incremental addition of Zn-Met. MZM and HZM groups demonstrated elevated amylase activity compared to the CON, with the HZM showing higher levels than the MZM. Carboxymethyl-cellulase exhibited significant linear changes (*p* < 0.001) upon Zn-Met supplementation. Compared to the CON treatment, carboxymethyl-cellulase activity had a significant linear improvement (*p* < 0.001) across all Zn-Met doses, with the MZM and HZM groups displaying higher levels than the LZM group. Supplementing the diet with Zn-Met enabled a linear enhancement of protease activity (*p* = 0.006). MZM and HZM groups showed higher protease levels than the LZM and CON groups.

### Ruminal fermentation characteristics

Table 1 showed that the supplementation of Zn-Met indicated a significant linear enhancement (*p* < 0.001) in a total concentration of total VFA. The average increases in total VFA were 6.59%, 9.57%, and 9.82% for the LZM, MZM, and HZM groups, respectively. The concentration of microbial protein exhibited significant linear changes (*p* = 0.009) upon Zn-Met supplementation. Microbial protein concentration rose linearly (*p* = 0.009) in the MZM and HZM treatments than in the CON group. However, this experiment did not yield significant evidence of treatment effects on pH, the molar proportions of each VFA, the C_2_:C_3_ ratio, and the NH_3_-N concentration (*p* > 0.05).

***Cumulative gas production and methane***

All dietary treatments improved gas production at 2 h post-incubation, with both linear (L) and quadratic (Q) effects being more significant (*p* = 0.009 and *p* = 0.006, respectively) than the CON group (Table 2). At 4 h after incubation, the MZM group exhibited the highest gas production in a quadratic manner (*p* = 0.015). When compared to the CON treatment, the LZM, MZM, and HZM groups demonstrated increased gas production at 6 h “(L; *p* = 0.002, Q; *p* < 0.001), 8 h (L; *p* = 0.002, Q; *p* < 0.001), 12 h (L; *p* = 0.003, Q; *p* < 0.001)”, and 48 h after incubation (L; *p* < 0.001, Q; *p* < 0.001). The gas production in all doses of dietary Zn-Met exhibited a linear enhancement (*p* < 0.001) at 24 h and a quadratic enhancement (*p* < 0.001) at 36 h after incubation. However, no statistically significant changes in CH_4_ production were discovered in the study (*p* > 0.05).

**Figure 2.**
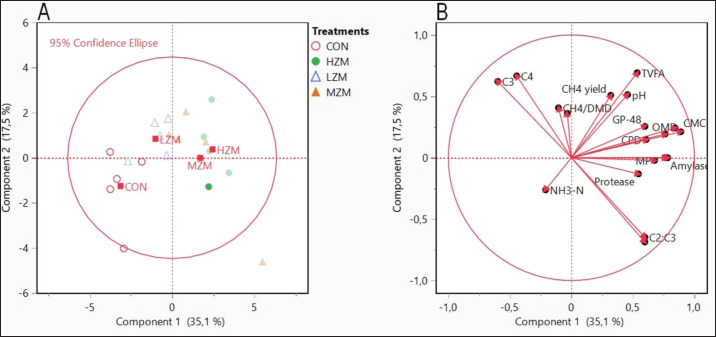
*In vitro* ruminal fermentation parameters plot utilizing principal component analysis. (A) Score plot. (B) Loading plot. CON: a basal diet without Zn-Met supplementation, LZM: low Zn-Met (30 mg Zn/kg DM), MZM: medium Zn-Met (60 mg Zn/kg DM), and HZM: high Zn-Met (90 mg Zn/kg DM). Abbreviations were provided in Table 4.

**Table 1.** Dietary effects of Zn-Met on the *in vitro* characteristics of rumen fermentation.

Item	Treatment				SEM	*p-*value	
	CON	LZM	MZM	HZM		Linear	Quadratic
pH	6.62	6.64	6.66	6.65	0.01	0.091	0.268
TVFA (mM)	56.74^a^	60.53^b^	62.17^b^	62.31^b^	0.67	<0.001	0.069
C_2_ (%)	66.43	65.94	67.12	67.08	0.32	0.302	0.737
C_3_ (%)	21.12	21.16	20.50	20.51	0.24	0.282	0.976
C_4_ (%)	12.45	12.90	12.38	12.42	0.10	0.505	0.317
C_2_:C_3_	3.15	3.12	3.32	3.28	0.06	0.317	0.963
NH_3_-N (mg/ml)	35.20	36.25	34.70	35.38	0.32	0.727	0.778
Microbial protein (mg/ml)	0.11^a^	0.15^ab^	0.16^b^	0.18^b^	0.01	0.009	0.503

Description: CON: a basal diet without Zn-Met supplementation, LZM: low Zn-Met (30 mg Zn/kg DM), MZM: medium Zn-Met (60 mg Zn/kg DM), HZM: high Zn-Met (90 mg Zn/kg DM), TVFA: total volatile fatty acids, C_2_: acetate, C_3_: propionate, C_4_: butyrate, C_2_:C_3_: acetate to propionate ratio, NH_3_-N: ammonia-nitrogen, SEM: standard error of the mean. Superscript letters on the same row indicate statistically significant differences at a significance level of *p* < 0.05.

### In vitro nutrient digestibility

Table 3 displayed the impact of various Zn-Met dosage levels on ruminal nutrient digestibility. According to the results, both the MZM and HZM revealed a linear enhancement (*p* < 0.001) in DM and OM digestibilities when compared to the CON. Additionally, the MZM and HZM groups presented a significant linear enhancement (*p* < 0.05) of the digestibility of CP and CF after 48 h of incubation.

### Principal component (PCs) analysis

Visualizing the relationships between Zn-Met dosage and the evaluated parameters was performed using PCA. A total of 19 variables, including microbial enzyme activities, rumen fermentation characteristics, gas production, and nutrient digestibility, were used for the analysis. These results revealed that the first four PCs calculated for more than 78.62% of the total information, as shown in Table 4. Regarding the total variance, these four PCs explained 35.12% (eigenvalue = 6.68), 17.54% (eigenvalue = 3.34), 16.43% (eigenvalue = 3.12), and 9.52% (eigenvalue = 1.81), respectively.

The score plot (Fig. 2A) utilized for the classification of Zn-Met supplementation demonstrated distinct separation based on dose. The CON and LZM groups were mainly positioned in the negative range along PC1, while the MZM and HZM groups were situated in the positive field, far from the CON group. Along PC2, the MZM and HZM groups were positioned in the positive range, while the CON group was in the opposing field. The MZM group aligned with the line range along PC2. The loading plot (Fig. 2B), which illustrates the relationship between variables, indicated that PC1 was primarily characterized by variables such as microbial protein, amylase, OM digestibility, gas production at 48 h, pH, CH_4_ yield, total VFA, C_2_:C_3_ ratio, and acetate levels on the right side. Additionally, variables such as NH_3_ levels, CH_4_ production per digested DM, C_4_, and C_2_ levels were prominent on the left side.

**Table 2.** Dietary effects of Zn-Met on the *in vitro* cumulative gas production and methane.

Item	Treatment				SEM	*p-*value	
	CON	LZM	MZM	HZM		Linear	Quadratic
Gas production (ml/300 mg DM) at							
2 h	0.95^a^	12.56^b^	2.96^b^	12.31^b^	0.24	0.008	0.006
4 h	8.82^a^	20.03^ab^	20.69^b^	19.48^ab^	0.26	0.141	0.015
6 h	4.10^a^	6.45^bc^	7.26^c^	25.94^b^	0.34	0.002	<0.001
8 h	9.86^a^	2.30^bc^	3.17^c^	31.79^b^	0.35	0.001	<0.001
12 h	9.37^a^	1.98^bc^	3.18^c^	41.47^b^	0.41	0.003	<0.001
24 h	9.05^a^	63.26^b^	4.45^b^	62.87^b^	0.55	0.001	0.923
36 h	9.70^a^	75.24^b^	5.89^b^	74.46^b^	0.67	0.367	<0.001
48 h	8.15^a^	83.67^b^	4.28^b^	83.06^b^	0.67	0.001	<0.001
CH_4_ (ml)	10.26	11.32	10.87	11.34	0.27	0.273	0.571
CH_4_/digested DM (ml/g)	74.67	80.51	70.41	76.50	1.85	0.728	0.985
CH_4_/digested OM (ml/gm)	89.18	94.06	85.80	92.08	2.39	0.974	0.917

Description: CON: a basal diet without Zn-Met supplementation, LZM: low Zn-Met (30 mg Zn/kg DM), MZM: medium Zn-Met (60 mg Zn/kg DM), HZM: high Zn-Met (90 mg Zn/kg DM), *t* h: cumulative gas production at *t* h of incubation, CH_4_: methane production (ml), CH_4_/digested DM: methane production per digested dry matter, CH_4_/digested OM: methane production per digested organic matter, SEM: standard error of the mean. Superscript letters on the same row indicate statistically significant differences at a significance level of *p* < 0.05.

**Table 3.** Dietary effects of Zn-Met on the *in vitro* nutrient digestibility.

Item	Treatment				SEM	*p-value*	
	CON	LZM	MZM	HZM		Linear	Quadratic
DM digestibility (%)	52.37^a^	53.43^a^	58.94^b^	60.43^b^	0.91	<0.001	0.832
OM digestibility (%)	49.83^a^	51.42^ab^	54.39^bc^	56.72^c^	0.81	<0.001	0.754
CP digestibility (%)	46.95^a^	51.83^ab^	53.31^b^	56.53^b^	1.22	0.004	0.686
CF digestibility (%)	39.05^a^	41.09^ab^	42.37^b^	42.93^b^	0.51	0.003	0.380

Description: CON: a basal diet without Zn-Met supplementation, LZM: low Zn-Met (30 mg Zn/kg DM), MZM: medium Zn-Met (60 mg Zn/kg DM), HZM: high Zn-Met (90 mg Zn/kg DM), SEM: standard error of the mean. Superscript letters on the same row indicate statistically significant differences at a significance level of *p* < 0.05.

## Discussion

The supplementation of Zn-Met in this study resulted in increased microbial enzyme activities, including amylase, CMC-ase, and protease. This improvement in enzyme activities can be attributed to the various functions performed by Zn supplementation. In ruminants, Zn may positively affect the fermentation pattern in the rumen by directly influencing microbial enzyme activity. Zn is involved in cell division, proliferation, and DNA synthesis. Another possible reason could be the role of Zn in enhancing the enzyme activities involved in the antioxidant properties [17]. These findings in the current research align with the previous study by Wang et al. [18], which reported increased CMC-ase activity following Zn supplementation in cows. Zn addition could enhance protease activity, positively linked with increased protein-degrading bacteria. Overall, this study and prior work collectively suggest that Zn-Met supplementation has the potential to favorably impact microbial enzyme activities within the rumen, supporting the critical role adequate Zn provision plays in facilitating optimal ruminal function and nutrient use among ruminant livestock.

**Table 4.** Eigenvalues of each principal component.

Variables	Principal component			
	1	2	3	4
Amylase	0.526	0.261	0.562	-0.197
CMC-ase	0.657	0.219	0.592	0.101
Protease	0.810	0.160	-0.139	-0.210
pH	0.184	-0.062	0.596	0.447
TVFA	0.646	-0.388	0.452	0.232
C_2_	0.164	0.978	0.060	0.042
C_3_	-0.246	-0.934	0.014	-0.130
C_4_	0.067	-0.837	-0.215	0.174
C_2_:C_3_	0.224	0.951	0.000	0.101
NH_3_-N	0.282	0.083	-0.814	0.098
MP	0.727	0.205	0.154	-0.100
GP-48	0.767	0.054	0.042	0.299
CH_4_yield	0.095	0.081	0.287	0.928
CH_4_/DMD	-0.092	-0.029	-0.158	0.944
CH_4_/DOM	-0.044	0.045	-0.150	0.946
DMD	0.471	0.194	0.767	0.063
OMD	0.408	0.121	0.784	-0.145
CFD	0.521	0.319	0.461	-0.025
CPD	0.622	0.075	0.256	0.005

Description: amylase: amylase enzyme activity, CMC-ase: carboxymethyl-cellulase enzyme activity, protease: protease enzyme activity, pH: pH value of rumen fluid, TVFA: total volatile fatty acids, C_2_: acetate, C_3_: propionate, C_4_: butyrate, C_2_:C_3_: acetate to propionate ratio, NH_3_-N: ammonia-nitrogen, MP: microbial protein, GP-48: gas production at 48 h of incubation, CH_4_ yield: methane production (ml), CH_4_/DMD: methane per digested dry matter, CH_4_/OMD: methane per digested organic matter, DMD: dry matter digestibility, OMD: organic matter digestibility, CFD: crude fiber digestibility, CPD: crude protein digestibility.

The result observed on ruminal pH values, ranging from 6.64 to 6.66, falls within the normal range for rumen fermentation, as Reis et al. [19] suggested. The NH_3_-N concentrations observed in all treatment groups in this study (ranging from 34.70 to 36.25 mg/ml) were within the typical biological range, as stated by McDonald et al. [20]. The similar NH_3_-N concentrations observed among the dietary groups in this study suggest that proteolysis and amino acids deamination processes were followed by increased assimilation of NH_3_-N into microbial biomass. This condition is further supported by the higher microbial protein levels observed in the treatments. Additionally, it could suggest similar activity levels of ammonia-producing bacteria. The variations observed in ruminal NH_3_-N levels across experiments might be ascribed to various variables, such as the differences in the ration’s formula and content, Zn levels, Zn sources, and potential interactions with other minerals [21].

Gas production is an exhaustive marker reflecting the extent of fermentation in the rumen. The positive correlation between gas production and the activity of ruminal microbes is widely recognized [22]. In the present study, gas production improved starting from 2 h after incubation with Zn-Met supplementation. However, Chen et al. [23] reported that gas production increased 9 h after incubation. Furthermore, cumulative gas production at 48 h was higher with Zn-Met supplementation. The lack of effect of CH_4_ production by Zn-Met supplementation in our investigation was aligned with the findings conducted by Fellner et al. [24], who described similar CH_4_ production between control and Zn inclusion in the form of HiZox, but a significant decrease with the addition of ZnO. Costa et al. [25] explained that Zn supplementation may increase the ruminal C_3_ concentration, which is an essential pathway for hydrogen competition. However, Zn-Met addition did not impact the C_3_ proportions in this study. The results of Zn supplementation in ruminant feed can vary depending on Zn’s chemical form, diet composition, and interactions between Zn and other dietary components.

The diets supplemented with Zn showed slightly higher *in vitro* ruminal VFA levels than the CON diet, which could be attributed to the increased microbial enzyme activity and protein in these diets. The enhanced microbial protein production and subsequent increase in VFA production indicate the beneficial impact of dietary Zn on the activity of the rumen microorganism. The culmination of carbohydrate fermentation by microbes produces VFA, which serves as a primary origin of metabolizable energy for ruminants. This finding aligns with the study by Shakweer [26], which described that adding inorganic and organic forms of Zn to the diet increased total VFA production.

As noted in this study, the enhanced digestibility of DM, OM, CF, and CP can be linked to the stimulative impact of Zn-Met supplementation on nutrient degradation within the rumen. This phenomenon is corroborated by the higher concentrations of total VFA and microbial enzyme activities observed across the Zn-treated groups comparable with the CON.

According to the PCA plot, the microbial enzyme activities were positioned close to nutrient digestibility, which was positioned on the right side of the x-axis (PC1). The elevated enzyme activities and enhanced functioning of microorganisms within the rumen probably contributed to the increased nutrient digestibility seen in the Zn-supplemented groups [27]. The negatively charged microbes interact with the positively charged Zn, creating a connection that aids in the attachment of microbes to the feed particles. This close association between microbes and feed particles facilitates the breakdown of complex compounds, allowing for efficient fermentation and nutrient degradation [28]. Previous research has also noted the favorable impacts of dietary Zn on the digestibility of nutrients. Garg et al. [29] stated that the digestibility of fiber in lamb was increased with Zn-Met addition in a basal diet, leading to improved lamb growth rates. Including Zn has been found to positively impact the breakdown and utilization of feed components in the rumen, resulting in enhanced nutrient digestibility [30]. This study established the effectiveness of utilizing Zn-Met as an organic source of Zn for rumen fermentation conditions *in vitro*. Nonetheless, further research is necessary to ascertain its impact on animals with Zn deficiency in their diet through *in vivo* experimentation.

## Conclusion

Based on our *in vitro* study, supplementing 60 and 90 mg Zn per kg DM as Zn-Met (organic source) in diets could improve rumen enzyme activities, fermentation characteristics, and nutrient digestibility without affecting methane production.

## List of abbreviations

AAS, atomic absorption spectroscopy; BW, body weight; C, celsius; C_2_, acetate; C_3_, propionate; C_4_, butyrate; CF, crude fiber; CMC-ase, carboxyl methyl cellulase; CO_2_, carbon dioxide; CON, a basal diet without Zn-Met supplementation; CP, crude protein; DM, dry matter; DNA, deoxyribonucleic acid; h, hour; HZM, high Zn-Met; CH_4_, methane; LZM, low Zn-Met; MZM, medium Zn-Met; NH_3_-N, ammonia-nitrogen; OM, organic matter; PCA, Principal Component Analysis; PCs, principal components; ppm, part per million; SAS, Statistical Analysis System; TDN, total digestible nutrient; v/v, volume per volume; VFA, volatile fatty acids; Zn-Met, Zn-methionine; Zn, zinc.
